# The Dark Triad Traits from a Life History Perspective in Six Countries

**DOI:** 10.3389/fpsyg.2017.01476

**Published:** 2017-08-30

**Authors:** Peter K. Jonason, Joshua D. Foster, Marina S. Egorova, Oksana Parshikova, Árpád Csathó, Atsushi Oshio, Valdiney V. Gouveia

**Affiliations:** ^1^School of Social Sciences and Psychology, Western Sydney University, Penrith NSW, Australia; ^2^Psychology, University of South Alabama, Mobile AL, United States; ^3^Lomonosov Moscow State University Moscow, Russia; ^4^University of Pécs Pécs, Hungary; ^5^Waseda University Shinjuku, Japan; ^6^Universidade Federal da Paraiba João Pessoa, Brazil

**Keywords:** narcissism, psychopathy, Machiavellianism, Dark Triad, life history theory, future consequences

## Abstract

Work on the Dark Triad traits has benefited from the use of a life history framework but it has been limited to primarily Western samples and indirect assessments of life history strategies. Here, we examine how the Dark Triad traits (i.e., psychopathy, Machiavellianism, and narcissism) relate to two measures of individual differences in life history strategies. In Study 1 (*N* = 937), we replicated prior observed links between life history strategies, as measured by the Mini-*K*, and the Dark Triad traits using samples recruited from three countries. In Study 2 (*N* = 1032), we measured life history strategies using the Consideration of Future Consequences Scale and correlated it with the Dark Triad traits in samples recruited from three additional countries. While there was some variability across participants’ sex and country, the results were generally consistent in that psychopathy and (to a lesser extent) Machiavellianism were related to faster life history strategies and narcissism was related to slower life history strategies. These results add cross-cultural data and the use of two measures of life history speed to understand the Dark Triad traits from a life history perspective.

## Introduction

There has been recent interest in the Dark Triad traits (i.e., psychopathy, narcissism, and Machiavellianism; Paulhus and Williams, 2002) to compliment most work in personality psychology on the Big Five traits (Furnham et al., 2013). One reason for this might be the integration of the traits into a life history perspective (see Wilson, 1975) which describes personality as reflecting *fast* life history strategies (i.e., *r-*selected, focused on multiple matings and immediate outcomes) and *slow* life history strategies (i.e., *K-*selected, focused on survival, investment in offspring, and immediate outcomes). For example, the Dark Triad traits are associated with a short-term mating strategy (Jonason et al., 2009), impulsivity (Jones and Paulhus, 2011), interpersonal aggression (Jones and Olderbak, 2014), and limited empathy (Jonason et al., 2013), all of which may reflect individual differences in life history strategies. These traits might facilitate the active exploitation of one’s socioecologies for the immediate extraction of sexual and survival benefits (Mealey, 1995). This is in contrast to traditional approaches to these traits as social pathologies. In this study, we attempt to add new and better detail to how the Dark Triad traits are correlated with life history indicators.

The Dark Triad traits are characterized by grandiosity and self-centeredness (i.e., narcissism), manipulation and cynicism (i.e., Machiavellianism), and callous social attitudes and impulsivity (i.e., psychopathy). Most work examining the relationship between the Dark Triad traits and life history strategies has been limited in a few ways. First, much of the work has assessed the association by examining purported, correlated effects like limited self-control (Jonason and Tost, 2010) or short-term mating (Jonason et al., 2009) and, more rarely, direct assessment of cognitive/motivational/affective/perceptual biases that may capture life history like, perhaps, a lack of consideration of future consequences (e.g., Strathman et al., 1994). Second, the research on the Dark Triad traits and life history strategies tends to rely too heavily on single, Western samples (but see Jonason et al., 2013), despite the utility of cross-cultural work in supporting evolutionary hypotheses. And third, the research, all too often assumes that the traits act in a uniform fashion in relation to life history predictions despite evidence to the contrary. For example, it appears to be psychopathy, in particular, that is a fast life history strategy (Jonason et al., 2010). Narcissism is associated with slow and fast life history traits when examining lower-order facets of each trait (McDonald et al., 2012), while Machiavellianism might be a slow life history strategy (Jones and Paulhus, 2009). Therefore, in this study we correlate the Dark Triad traits with individual differences in life history strategies as captured by the Mini-*K* (Study 1; which replicates Jonason et al., 2010) and the consideration of future consequences (Study 2) in six countries.

We make predictions for each of the traits given the modest correlations among the traits (Furnham et al., 2013). First, psychopathy has the strongest and most reliable association with a fast life history strategy (Mealey, 1995; Jonason et al., 2017b). As such, we expect it to be associated with a fast life history strategy in relation to both Mini-*K* scores and the consideration of future consequences. Second, evidence for the life history pattern associated with Machiavellianism is unclear because (1) it might be redundant to psychopathy (McHoskey et al., 1998; Persson et al., 2017), (2) it might be associated with long-term planning (Jones and Paulhus, 2009) which is a slow trait, and (3) it is characterized by antigroup group values which might be evidence of its fast life history strategy (Jonason et al., 2015). We expect that Machiavellianism will be associated with a fast life history strategy, but only to the extent that it shares variance with psychopathy. When controlling for psychopathy, we do not expect Machiavellianism to be uniquely associated with a fast life history strategy. Third, the relationship between narcissism and life history indicators is also unclear. It is often associated with fast life history traits in terms of short-term mating (Jonason et al., 2009) but slow life history traits like prosocial values (Jonason et al., 2015). We predict that narcissism will be associated with a *slow* life history strategy when life history strategy is measured with the Mini-*K* and individual differences in the consideration of future consequences because we contend that narcissism has its *fast* origins in mating only and otherwise, is somewhat *slow* because being so affords narcissists the kinds of social connections that possess long-term utility. We test these predictions in two studies, drawn from six countries, testing for the stability of these correlations across each country and in men and women. We expect limited moderation by country as we see the Dark Triad traits as universal adaptations to local ecological conditions (Jonason and Schmitt, 2017) and minor moderation by participants’ sex such that correlations between the Dark Triad traits and life history strategy markers should evidence that men are particularly *fast* and women are particularly *slow* (Figueredo et al., 2005).

One of the reasons the Dark Triad traits have become so interesting in popular and research circles is their integration into a life history framework. Unfortunately, that research has a few limitations that we hope to address here. In this study, we examine how the Dark Triad traits are correlated with individual differences in the speed of someone’s life history strategies with two measures (i.e., the Mini-*K* and the Consideration with Future Consequences Scale) in samples drawn from America, Australia, Brazil, Hungary, Japan, and Russia. Such tests should provide new and improved tests of how one can use life history theory to understand these darker aspects of personality.

## Materials and Methods

### Participants and Procedures

We present here analyses from anonymous, online, undergraduate students who were remunerated with course credit and previously reported in Jonason et al. (2017a) who took part in this study in an unsupervised manner. **Study 1** was composed of 300 Hungarian (129 men), 306 Brazilian (91 men), and 331 American (90 men) undergraduates (*M_Age_* = 22.67, *SD_Age_* = 4.66, *Range* = 18 to 47). **Study 2** was composed of 310 Australian (97 men), 351 Japanese (135 men), and 371 Russian (94 men) undergraduates (*M_Age_* = 20.13, *SD_Age_* = 2.77, *Range* = 16 to 45). Each author received ethics approval at their respective ethics review board. Where measures were not in English or have not yet been validated in the country-specific language (e.g., Japan; Shimotsukasa and Oshio, 2017), the items were translated by one native speaker, back translated by another, and then checked by a native English speaker. Participants took part in a larger online study about “personality and views of the future” where they first provided consent through a tick-box, proceeded to complete the study, and were thanked and debriefed upon completion.

### Measures

Both samples took the 27-item Short Dark Triad (Jones and Paulhus, 2014), which was used to measure Machiavellianism (e.g., “I like to use clever manipulation to get my way.”), narcissism (e.g., “I insist on getting the respect I deserve.”), and psychopathy (e.g., “People who mess with me always regret it.”). Participants indicated their agreement to the above with (1 = *strongly disagree*; 5 = *strongly agree*). Items for each scale were averaged together to create indexes of narcissism (α’s = 0.51 to 0.79), Machiavellianism (α’s = 0.59 to 0.77), and psychopathy (α’s = 0.60 to 0.78).^1^^,^^2^

The sample from **Study 1** completed the Mini-*K* (Figueredo et al., 2005). It is a 20-item measure of life history strategy, by indicating how much they agreed (1 = *not at all*; 5 = *very much*) with a series of such statements as “I can often tell how things will turn out” and “I avoid taking risks.” We averaged these items to create an overall index of each individual’s life history strategy (α’s = 0.76 to 0.83). The Mini-*K* was scored such that larger values indicated a slower life history strategy.^3^

The sample from **Study 2** completed the Consideration of Future Consequences Scale (Strathman et al., 1994). It is composed of 12 items where participants were asked how characteristic of them (1 = *extremely uncharacteristic*; 5 = *extremely characteristic*) statements like “I consider how things might be in the future, and try to influence those things with my day to day behavior” and “Often I engage in a particular behavior in order to achieve outcomes that may not result for many years.” Items were averaged to create an index of individual differences in the consideration of future consequences (α’s = 0.66 to 0.67) where higher scores indicated more concern for the future (or a slower life history strategy).^4^

## Results

In **Table 1**, we correlated the Dark Triad traits with the Mini-*K* (Study 1) and the Consideration of Future Consequences Scale (Study 2) overall, across sample-sites, and in men and women with a more conservative *p*-value (0.01) given the large number of comparisons. When looking at data from all three countries, we found that Machiavellianism and psychopathy embodied a *fast* life history strategy as seen in individual differences in the Mini-*K* and the Consideration of Future Consequences whereas narcissism embodied a *slower* approach as measured in the same way. While there was some variability in these results across country, biological sex, and method of assessment (see **Table 1** for contrasts), these differences were overshadowed by the general trends observed. We had only one case of moderation by country, suggesting the correlation between narcissism was negative in Australia but positive in Japan; an effect that appears localized to women.

**Table 1:** Correlations between the Dark Triad traits and life history strategies overall and in six countries (Study 1/Study 2), in men and women, and within-trait, across countries.

	Study 1 (*N* = 937)				Study 2 (*N* = 1032)			
		
	Mini-*K*				Consideration of future consequences			
		
Overall	Overall	Men	Women	*z*	Overall	Men	Women	*z*
Machiavellianism	-0.22^∗∗^	-0.16^∗∗^	-0.20^∗∗^	0.62	-0.13^∗∗^	-0.01	-0.17^∗∗^	2.34
Narcissism	0.18^∗∗^	0.19^∗∗^	0.21^∗∗^	-0.31	0.12^∗∗^	0.10	0.13^∗∗^	-0.44
Psychopathy	-0.37^∗∗^	-0.38^∗∗^	-0.32^∗∗^	-1.02	-0.27^∗∗^	-0.21^∗∗^	-0.29^∗∗^	1.24

**America/Australia**	*n* = 331	*n* = 90	*n* = 241		*n* = 310	*n* = 97	*n* = 213	

Machiavellianism	-0.25^∗∗^	-0.27^∗^	-0.22^∗^	-0.43	-0.13	0.05	-0.18	1.87
Narcissism	0.17^∗^	0.06	0.22^∗^	-1.31	-0.13	-0.05	-0.12	0.57
Psychopathy	-0.38^∗∗^	-0.51^∗∗^	-0.30^∗∗^	-2.02	-0.29^∗∗^	-0.22	-0.29^∗∗^	0.60

**Brazil/Japan**	*n* = 306	*n* = 91	*n* = 208		*n* = 351	*n* = 135	*n* = 216	

Machiavellianism	-0.12	-0.17	-0.11	-0.48	0.03	0.03	0.03	0.00
Narcissism	0.14	0.22	0.12	0.81	0.16^∗^	0.14	0.17^∗^	-0.28
Psychopathy	-0.27^∗∗^	-0.26	-0.28^∗∗^	0.17	-0.13	-0.25^∗^	-0.07	-1.67

**Hungary/Russia**	*n* = 300	*n* = 129	*n* = 171		*n* = 371	*N* = 94	*n* = 277	

Machiavellianism	-0.21^∗∗^	-0.01	-0.29^∗∗^	2.45	-0.07	-0.05	-0.09	0.33
Narcissism	0.21^∗∗^	0.26^∗∗^	0.23^∗^	0.27	0.06	0.03	0.07	-0.33
Psychopathy	-0.38^∗∗^	-0.28^∗∗^	-0.39^∗∗^	1.05	-0.18^∗^	-0.04	-0.25^∗∗^	1.78

*Country comparisons* (*z*)	*America → Brazil*				*Australia → Japan*			

Machiavellianism	-1.69	-0.70	-1.19		-2.05	0.15	-2.18	
Narcissism	0.39	-1.08	1.08		-3.73^∗∗^	-1.42	-3.01^∗∗^	
Psychopathy	-1.55	-1.96	-0.23		-2.14	0.24	-2.24	

	*America → Hungary*				*Australia → Russia*			

Machiavellianism	-0.53	-1.91	0.74		-0.78	0.68	-1.00	
Narcissism	-0.52	-1.48	-0.11		-2.47	-0.54	-2.08	
Psychopathy	0.00	-1.97	1.02		-1.51	-1.25	-0.47	

	*Brazil → Hungary*				*Japan → Russia*			

Machiavellianism	1.13	-1.16	1.81		1.34	0.59	1.32	
Narcissism	-0.89	-0.31	-1.09		1.36	0.81	1.11	
Psychopathy	1.51	0.16	1.19		0.69	-1.58	2.03	


**z* is Fisher’s *z* to compare independent correlations. ^∗^*p* < 0.01, ^∗∗^*p* < 0.001.*

Given that the Dark Triad traits were correlated, we used standard multiple regression to determine if the Dark Triad traits (entered together) accounted for unique variance in scores on the Mini-*K* (Study 1) and Consideration of Future Consequences Scale (Study 2). Together, the Dark Triad traits predicted scores on the Mini-*K* overall [*R* = 0.49, *F*(3,929) = 96.48, *p* < 0.01] and, when we disaggregated the sample, in the American [*R* = 0.48, *F*(3,327) = 33.28, *p* < 0.01], Hungarian [*R* = 0.52, *F*(3,296) = 37.04, *p* < 0.01], and Brazilian [*R* = 0.36, *F*(3,298) = 15.16, *p* < 0.01] samples. However, whereas psychopathy and narcissism each accounted for significant unique variance in scores on the Mini-*K* in all samples, Machiavellianism did not account for unique variance in any of the samples. Likewise, the Dark Triad traits together predicted scores on the Consideration of Future Consequences Scale overall [*R* = 0.31, *F*(3,1028) = 31.92, *p* < 0.01] and, when we disaggregated the sample, in the Australian [*R* = 0.29, *F*(3,306) = 9.53, *p* < 0.01], Russian [*R* = 0.20, *F*(3,367) = 5.33, *p* < 0.01], and Japanese [*R* = 0.29, *F*(3,347) = 10.20, *p* < 0.01] samples. Psychopathy uniquely and *negatively* predicted consideration of future consequences in all samples, while narcissism uniquely and *positively* predicted the same in the Japanese sample. Machiavellianism did not account for unique variance in any of the samples. In sum, psychopathy and narcissism showed evidence of being unique predictors of fast and slow life history strategies, respectively. Machiavellianism, in contrast, did not uniquely predict fast life history beyond variance already accounted for by psychopathy.

## Discussion

Studying the Dark Triad traits using a life history paradigm has been incredibly fruitful. However, it has some limitations that we have attempted to address here by (1) directly testing the relationship between the Dark Triad traits and two life history biases as measured with the Mini-*K* and the Consideration of Future Consequences Scale, (2) adopting a multinational sample, (3) better distinguishing each trait’s unique relationship with life history traits. Our study revealed three findings that were generally stable across country and participants’ sex. First, psychopathy was linked to a fast life history speed and a failure to consider future consequences, thereby affirming its position as the part of the Dark Triad that can be best labeled as a “fast” trait (Mealey, 1995). Second, Machiavellianism appears to be superficially and spuriously associated with life history speed and the consideration future consequences. It may be redundant to psychopathy (Persson et al., 2017) or it may be that the removal of shared variance results in fast and slow aspects of Machiavellianism canceling themselves out in our assessment (Jonason et al., 2012b). And third, narcissism was associated with a slow—not a fast—life history pattern as seen in high scores on the Mini-*K* and a tendency to consider future consequences. This slow bias might be (1) because narcissism is genuinely slow, (2) driven by the measure used that does not allow for facet-level analyses which might be important (McDonald et al., 2012), or (3) narcissism might only be fast in relation to sex and relationships but not globally so (Jonason et al., 2009, 2015). Future work is called for to better understand (a) where, when, and why Machiavellianism appears to be redundant to psychopathy in some contexts but not others and (b) the relationship between narcissism and life history traits. For example, Machiavellianism might be redundant when accounting for sexual and romantic motivations (Jonason et al., 2012a), but important when accounting for the deployment of manipulative social strategies (Jonason et al., 2012b).

Although the effects were weak, we did find some moderation by participants’ sex of the correlations consistent with life history models of the Dark Triad traits. We found that women who were particularly low on Machiavellianism were also particularly *slow*, more so than men who were low on the trait. In contrast, we found that men who were particularly high on psychopathy were also particularly *fast*, more so than women who were high on the trait. This may be consistent with the idea that these traits are more adaptive (i.e., reproductive benefits > reproductive costs) for men than women. However, it must be noted that these did not present themselves consistently across countries and thus, we urge caution in their interpretation. Similarly, we only found weak evidence of moderation by country. Nevertheless, one notable pattern suggests that the Australian and Japanese samples differed in how the Dark Triad traits were linked with the consideration of future consequences (i.e., Study 2). This might be a function of country-level differences in individualism and collectivism as the benefits for delaying reward might be simultaneously beneficial to the group and the individual whereas, not delaying may be especially beneficial to the individual. Future work will need to better understand cross-cultural patterns associated with the Dark Triad traits.

## Conclusion

Despite the use of multinational data and two measures to capture individual differences in life history strategies, the present studies contained several limitations. First, although our samples were recruited from college students from various regions around the globe, they were limited to one Asian country, two Eurasian counties, one South American country, and two predominantly Anglo-Saxon countries, all, of which could all be described as educated, industrialized, rich, democratic (Henrich et al., 2010), and female. Future research should include a more diverse set of cultures and participants to more firmly establish the generalizability of the associations reported in this paper.

Second, while most of the measures used in this research passed the standard threshold for internal consistency (i.e., 0.70; Nunnally, 1978), a few only passed the more liberal threshold set out for basic research (i.e., 0.50; Schmitt, 1996). However, the scales in the full datasets in each study had sufficient internal consistency making only the country-specific associations slightly less trustworthy. A related point of concern might be that we had to use *ad hoc*, translated measures in some of our samples. However, as our results line-up across countries, we feel they are reasonably trustworthy. Despite this, our results would be bolstered if we included validity or response checks. Future research would benefit from the use of measures with stronger psychometric properties regardless of the culture/language they are administered in.

Third, we used relatively short measures (which might have suppressed internal consistency) throughout, which prohibit more fine-grained analyses, such as analyses of narcissism’s putative sub-facets (Foster et al., 2015) or the multidimensional nature of life history batteries (Jonason et al., 2013). Such analyses might reveal that the lower-order facets of each trait in the Dark Triad traits accounts for unique and even apparently contradictory information about the life history model of the Dark Triad traits (McDonald et al., 2012). However, as the traditional content of the Dark Triad traits was atheoretically derived and not compiled in reference to the other traits, there is potentially irrelevant content in each trait as it is measured.

Fourth, our data were correlational, cross-sectional, and self-report in nature making causal claims highly speculative. For example, we cannot be certain whether personality, life history strategies, or something else operate as the causal factor given the present data. Future research would benefit from the use of experimental and longitudinal designs to clarify these issues. For example, experiments that manipulate socioecological contingencies and determine if those high in the Dark Triad traits experience a shift toward *fast*/*slow* life history strategies should be conducted. Importantly, methods should be adopted that move life history studies on the Dark Triad traits beyond self-report methods.

Despite these limitations, we have provided more robust tests of the relationship between the Dark Triad traits and life history strategies using two measures of the latter and data drawn from six countries. We hope this paper will encourage more detailed, rigorous, and critical assessments of the life history perspective on the Dark Triad traits. In short, this study demonstrated different life history biases in psychopathy and narcissism, and to a lesser extent, Machiavellianism around the world.

## Authors’ Note

Data reported here were previously reported in Jonason et al. (2017a).

## Ethics Statement

This study was carried out in accordance with the recommendations of the APA and the Human Ethics committee at Western Sydney University, the University of South Alabama, University of Pics, Lomonosov Moscow State University, Waseda University, and Universidade Federal da Paraiba with online, tick-box informed consent from all subjects. All subjects gave written informed consent in accordance with the Declaration of Helsinki. The protocol was approved by the Human Ethics committee at the aforementioned university ethics boards.

## Author Contributions

The primary author is responsible for the design, analyses, and writing of this paper. The second author provided feedback on an earlier version of the paper and the remaining authors were responsible for collecting data in their respective countries.

## Conflict of Interest Statement

The authors declare that the research was conducted in the absence of any commercial or financial relationships that could be construed as a potential conflict of interest.

## Footnotes

1 In the full sample of Study 1, Machiavellianism was correlated with narcissism (*r* = 0.24, *p* < 0.01) and psychopathy (*r* = 0.55, *p* < 0.01) and psychopathy was correlated with narcissism (*r* = 0.32, *p* < 0.01). These correlations held up across the different samples. In the full sample for Study 2, Machiavellianism was not correlated with narcissism (*r* = -0.02) but it was correlated with psychopathy (*r* = 0.46, *p* < 0.01) and psychopathy was correlated with narcissism (*r* = 0.15, *p* < 0.01). The three were better correlated in the Australian (*r*s = 0.34 to 0.55) and the Russian (*r*s = 0.15 to 0.44) samples but less so in the Japanese sample (*r*s = -0.05 to 0.41).

2 Cronbach’s alphas were the lowest in Brazil for each of the Dark Triad traits.

3 Only American [*t*(329) = -2.92, *p* < 0.01, Cohen’s *d* = -0.32] and Hungarian [*t*(298) = -3.63, *p* < 0.01, *d* = -0.42] women scored higher than men on the Mini-*K*.

4 Only Australian women compared to Australian men were more likely to consider future consequences [*t*(308) = -2.17, *p* < 0.05, *d* = -0.24].
